# Giant bladder stone resulting in acute kidney injury; A case report and review of literature

**DOI:** 10.1016/j.eucr.2022.101991

**Published:** 2022-01-07

**Authors:** Fitsum Gebreegziabher Gebrehiwot, Mubarek Bargicho Adem, Ibsa Kedir Hassen, Kaleab Habtemichael Gebreselassie, Ferid Ousman Mummed, Feysel Hassen Issack

**Affiliations:** Urology Unit, Department of Surgery, St. Paul's Hospital Millennium Medical College, Swaziland Street, Addis Ababa, Ethiopia

**Keywords:** A case report, Giant bladder stone, Open cystolithotomy, Acute kidney injury

## Abstract

A giant bladder stone is very rare in adults. We report a case of giant bladder stone causing acute kidney injury in a 23-year-old male, who presented with lower urinary tract symptoms (LUTS) characterized by both irritative and obstructive LUTS. In addition, he also had episodes of reddish urine for the past decade. A non-contrast-enhanced CT scan was used for the diagnosis. Open cystolithotomy was performed and a 500g weighing stone was removed. He developed a superficial surgical site infection which was treated with wound care. He was discharged improved. Improvement in symptoms and serum creatinine was noted on follow-up.

## Abbreviations

CT: Computed tomography
IV: Intravenous
LUTS: Lower urinary tract symptoms
STIs: Sexually transmitted infections

## 1 Introduction

A giant bladder stone, as defined in several kinds of literature, is a bladder stone weighing more than 100 g.^1^ It is a rare clinical entity in modern medical practice with very few cases reported so far.^2^ We report a case of giant bladder stone resulting in acute kidney injury in a 23-year-old male patient.

## 2 Case presentation

A 23 years old male patient from a rural part of Ethiopia was referred to a tertiary hospital in Addis Ababa and seen at a urology clinic. He presented with urinary urgency, frequency, and intermittent dysuria since childhood and intermittent hematuria of 10 years duration. The patient also developed bilateral flank pain, decreased urine volume, a sense of incomplete voiding, urinary intermittency, and dribbling that progressively worsened. There was no preceding trauma history. His sexual history is unremarkable for Sexually transmitted infections (STIs). Physical examination was evident for hypertension with multiple high blood pressure records. There was a mild suprapubic tenderness and the urethral catheter was inserted without difficulty and drained clear urine.

**Fig. 1:**
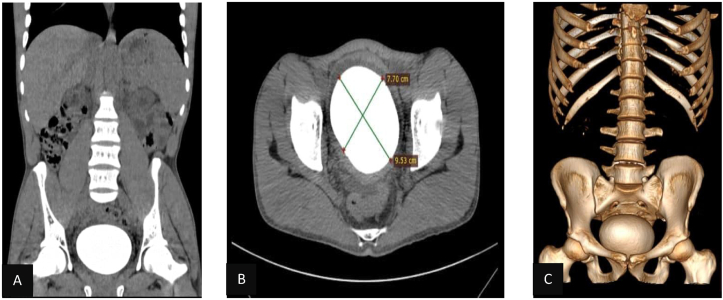
Pre-operative non-contrast CT of the abdomen and pelvis. A: Coronal Reconstruction B: Axial image C: 3D reconstruction.

The urinalysis was remarkable for blood (+3), leukocyte (+3), and a full microscopic field of bacteria and white blood cells. Complete blood count was normal. Serum creatinine level was raised to 4.1 mg/dl and a serum potassium measurement showed hyperkalemia (5.5 mEq/L). On imaging, abdominopelvic ultrasound showed evidence of moderate bilateral hydronephrosis as well as an 8.5 cm in length bladder stone with chronic bladder wall changes. The prostate has normal size and volume. Furthermore, A non-contrast abdominopelvic CT scan revealed a large bladder stone, measuring 9.5*7.7cm, that has a round to oval shape (Fig. 1.) as well as bilateral moderate hydroureteronephrosis.

**Fig. 2:**
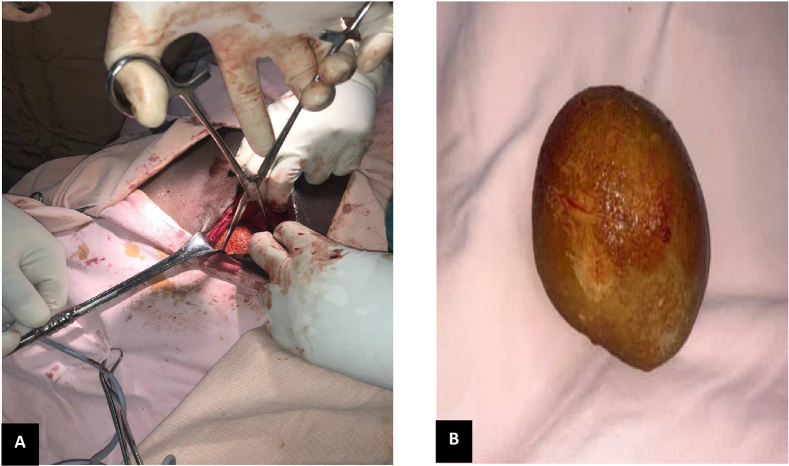
Intra-operative pictures. A: Stone being extracted with stone forceps B: Stone removed.

After Hyperkalemia is corrected and the UTI (urinary tract infection) is treated with intravenous (IV) antibiotics, the patient underwent an open Cystolithotomy. The stone removed weighed 500 g (Fig. 2).

The patient developed a superficial surgical site infection on the fifth postoperative day, which was treated with wound care and delayed primary closure. The patient was discharged improved on the 11th postoperative day. The urethral catheter was removed on the 14th post-operative day. On subsequent follow-up at the urology clinic, the LUTs resolved, urine volume normalized and blood pressure was within the normal range. The Serum creatinine level dropped to 1.3 mg/dL on the 10^th^ day postoperatively and subsequently normalized. Marked improvement of hydroureteronephrosis was also noted on ultrasound.

## 3 Discussion

A bladder stone weighing more than 100 g is a rare clinical entity in modern urologic practice. Patients with giant bladder stones often present with recurrent and long-standing lower urinary tract symptoms, hematuria, and urinary retention.^3^

Bladder calculi are most commonly seen in patients with BPH, urethral stricture, migration from the upper tract. The exact etiology, in our patient, is not identified. Nevertheless, the most likely cause was suspected to be recurrent urinary tract infection (UTI). There are about 86 cases ever reported in the English medical literature. The largest bladder stone ever removed weighs 1640 g.^4^

Most compositions of the bladder stones include triple phosphate, calcium carbonate, and calcium oxalate. Our patient's bladder stone composition was not analyzed because the stone analysis is not available in our setup. Very few reports of giant bladder stones presented with acute kidney injury.^5^

Although some mention Cystoscopy as the preferred method of diagnosis, plain abdominal x-ray, pelvic ultrasound, and CT scan, alone or in combination, were used to diagnose most giant bladder stones.

There are several available modalities and techniques mentioned in the standard textbooks to remove bladder stones. Almost all of the giant bladder stones reported in the literature were managed with open cystolithotomy.

## 4 Conclusion

We believe this case is interesting for two reasons. Our patient didn't have an infra-vesical obstruction that predisposed him to the giant bladder stone and also is among the rare patients with giant bladder stone that presented with acute kidney injury. However, we suspected recurrent UTI during childhood as a possible cause of the bladder stone. To the best of our knowledge, our patient represents one of the largest bladder stones ever removed and reported in this country.

## Ethics approval and consent to participate

Ethical clearance was obtained from the institutional review board of St. Paul's Hospital Millennium Medical College and is available to the editors upon request.

## Consent for publication

Informed written consent was obtained from the patient for the publication of this article. A copy of the written consent is available for review by the Editor-in-Chief of this journal upon reasonable request.

## Availability of data and material

The datasets with more images and patient data are available from the corresponding author on reasonable request.

## Funding

This manuscript did not receive any specific grant from funding agencies in the public, commercial or non-profit sectors.

## Authors' contributions

FHI, FOM, and KHG conceived the idea. FGG, and MBA, operated on the patient. FHI, FOM, KHG, and IKH wrote the draft. All authors contributed to, read, and approved the final manuscript.

## Declaration of competing interest

The authors declare that they have no competing interests.
